# Clinical efficacy of fecal microbiota transplantation in alleviating depressive symptoms: a meta-analysis of randomized trials

**DOI:** 10.3389/fpsyt.2025.1656969

**Published:** 2025-10-06

**Authors:** Xiaotao Zhang, Ying Li, Yueyue Guo, Jia Sun, Yang Yang

**Affiliations:** Department of Nursing, Jiangsu Province Hospital of Chinese Medicine, Affiliated Hospital of Nanjing University of Chinese Medicine, Nanjing, China

**Keywords:** depression, depressive symptoms, fecal microbiota transplantation, gut microbiota, systematic review, meta-analysis

## Abstract

**Background:**

Depressive symptoms are common in neuropsychiatric disorders, significantly affecting quality of life and posing challenges to treatment. While pharmacological and psychological therapies remain standard, many patients show limited response. Fecal microbiota transplantation (FMT), which aims to restore gut microbial balance, has emerged as a novel approach for alleviating depressive symptoms by modulating the gut-brain axis. This study aims to conduct a comprehensive synthesis and quantitative evaluation of current evidence to elucidate the therapeutic potential of FMT in the management of depressive symptomatology.

**Methods:**

Following PRISMA guidelines, we conducted a systematic search across PubMed, Embase, Web of Science, the Cochrane Library, and CINAHL from January 1, 2000, to December 31, 2024. 12 randomized controlled trials (RCTs) with 681 participants were included. The standardized mean difference (SMD) was calculated to evaluate FMT’s effect on depressive symptoms. Subgroup analyses examined effects by delivery routes, follow-up duration, and clinical population.

**Results:**

FMT significantly reduced depressive symptoms (SMD = -1.21; 95% CI: -1.87 to -0.55; *p* = 0.0003). Sensitivity analysis confirmed statistical significance (SMD = -0.56; 95% CI: -0.86 to -0.26; *p* = 0.001). Both oral capsule and direct gastrointestinal administration were effective, with greater effects seen in direct gastrointestinal delivery (SMD = -1.06 vs. -1.29). Improvements were most notable in the short- to mid-term; effects diminished by 6 months. Subgroup analysis showed stronger effects in patients with irritable bowel syndrome (IBS) (SMD = -1.06) than in those with neurological/psychiatric-related conditions (SMD = -0.67), with moderate heterogeneity (I² = 47%).

**Conclusions:**

This meta-analysis supports FMT as an effective adjunctive therapy for depressive symptoms, especially in individuals with IBS. Endoscopic or enema routes appear more efficacious than oral capsules. While short- and mid-term benefits are evident, sustained effects require further investigation through long-term, high-quality RCTs.

**Systematic review registration:**

https://www.crd.york.ac.uk/PROSPERO/, identifier CRD42025638185.

## 1 Introduction

Depressive symptoms, characterized by persistent negative mood, diminished interest, cognitive impairment, and sleep disturbances, constitute key features of various neuropsychiatric disorders (1). Such symptoms are frequently observed as comorbid conditions in chronic illnesses, with notably high prevalence rates across numerous patient populations (2, 3). According to data from the World Health Organization (WHO) in 2021, the number of individuals affected by depression globally exceeded 280 million (4). In China, the lifetime prevalence rate of depression was reported to be approximately 3.4%. Particularly concerning is the prevalence among adolescents, reaching as high as 14.8%, accompanied by emerging trends towards younger age at onset and increased incidence within occupational groups (5). Depressive states negatively affect patients’ adherence to chronic disease management protocols, exacerbating disease progression, elevating hospitalization rates, and consequently increasing healthcare expenditures (6). Moreover, depressive symptoms markedly impair both occupational and social functioning. Mood disturbances and cognitive deficits can substantially reduce workplace productivity, and in severe cases, result in total incapacitation. Survey data indicate that between 2010 and 2021, depressive disorders rose to become the second leading cause of years lived with disability (YLD) worldwide (7). This not only imposes significant economic burdens at the individual level but also entails considerable societal costs. Thus, managing depressive symptoms has emerged as a critical public health challenge, underscoring the urgent need for effective therapeutic strategies to mitigate their detrimental impact on both individuals and society.

Although pharmacotherapy and psychotherapy represent primary therapeutic modalities for depression, a significant proportion of patients experience inadequate symptom relief (8). Pharmacological treatments often induce adverse effects, while psychotherapies typically require substantial time and resource investments. Therefore, exploring novel, alternative therapeutic approaches is imperative to better accommodate the diverse needs of patients.

Recently, considerable attention has been devoted to the role of gut microbiota in modulating emotional and behavioral responses. Accumulating evidence supports the involvement of the microbiota-gut-brain (MGB) axis as a critical mediator of depressive symptoms (9–12). Despite variability in specific findings across individual studies, a common observation is the altered gut microbiota composition in patients with depression compared to healthy controls, characterized by an enrichment of pro-inflammatory bacterial species and reductions in anti-inflammatory microbiota populations, reinforcing the inflammatory hypothesis of depression (13–15). Beyond compositional shifts, gut microbiota influence emotional and behavioral states through bioactive metabolites, including short-chain fatty acids (SCFAs) (16–19), neurotransmitter-related molecules, and trimethylamine N-oxide (TMAO) (20). These microbial-derived metabolites play a pivotal role in mediating gut-brain communication via the microbiota-gut-brain axis (MGBA), a bidirectional pathway critical for regulating cognitive, emotional, and behavioral functions (21, 22).

Notably, disorders of gut-brain interaction (DGBIs), particularly irritable bowel syndrome (IBS), exemplify the clinical relevance of MGBA dysfunction. DGBIs are characterized by chronic gastrointestinal symptoms—such as pain, motility disturbances, dysbiosis, and immune activation—in the absence of structural abnormalities (23, 24). IBS exhibits one of the highest rates of psychiatric comorbidity among gastrointestinal disorders (25, 26), further implicating MGBA dysregulation in mood disorders. Given these findings, targeting gut microbiota represents a promising therapeutic strategy for depression, with potential mechanisms including modulation of inflammatory pathways, microbial metabolite production, and gut-brain signaling.

Fecal microbiota transplantation (FMT), an emerging therapeutic intervention, restores gut microbial homeostasis by transferring microbial communities from healthy donors into the recipient’s gastrointestinal tract, presenting a promising alternative treatment for depressive symptoms. Preclinical studies have demonstrated that fecal microbiota transplantation from individuals with major depressive disorder can induce depressive-like behavior in rodents, whereas transplanting microbiota from healthy donors may reverse these behavioral phenotypes (27, 28). Kurokawa et al. (29) observed reduced microbiota diversity in patients with Hamilton Depression Rating Scale (HAM-D) scores ≥ 8 compared to healthy donors and individuals score < 8. Following FMT treatment, an increase in microbial diversity correlated with symptom improvement. Similarly, Fang et al. (30) demonstrated that FMT significantly elevated the abundance of beneficial bacteria such as Lactobacillus, Bifidobacterium, Turicibacter, Anaerostipes, and Eisenbergiella, resulting in substantial alleviation of anxiety and depression in patients with chronic insomnia. Doll et al. (31) described two cases of treatment-resistant major depression responding to adjunctive FMT, with symptom relief within four weeks. More recently, Green et al. (32) initiated a triple-blind, randomized pilot trial evaluating enema-delivered FMT in Major Depressive Disorder (MDD), providing groundwork for future efficacy research.

Despite these encouraging findings, current evidence on FMT for depression is fragmented and inconclusive. A systematic review conducted in 2021, which encompassed 62 studies, confirmed the benefits of microbiota-targeted interventions such as probiotics, prebiotics, and synbiotics on depressive symptoms; however, only one study specifically evaluated FMT (33). In addition, Meyyappan et al. (34) conducted an earlier systematic review summarizing preclinical studies, case reports, and small-scale clinical trials on the impact of FMT across various psychiatric disorders, including depression, while they highlighted FMT’s promise across psychiatric disorders but did not perform a meta-analysis due to the heterogeneity and low quality of evidence. Recent researchers have conducted retrospective analyses of FMT for depression (11, 12, 35), but quantitative evaluations remain sparse, and inconsistencies persist owing to participant heterogeneity and limited sample sizes. Previous evidence regarding the impact of FMT on depressive symptoms remains limited. In a meta-analysis focused on patients with IBS, Wang et al. synthesized four randomized controlled trials in which depression was evaluated as a secondary outcome and reported no statistically significant difference between FMT and placebo at 12 or 24 weeks of follow-up (36). These results highlight the uncertainty surrounding the antidepressant efficacy of FMT, particularly when depressive symptoms are not the primary treatment target.

The safety, efficacy, and cost of FMT are collectively influenced by the choice of administration route. In clinical practice, commonly used FMT delivery routes include nasogastric or nasojejunal intubation, gastroduodenoscopic infusion, oral capsules, retention enemas, and colonoscopic administration. The FADDA study (37) reported that oral fecal microbiota capsules substantially improved patient uptake and acceptance, with fewer adverse effects and markedly superior efficacy compared to other administration routes. However, findings across diseases remain inconsistent. A 2023 meta-analysis (38) found no statistically significant differences in induction of remission rates for IBD among different administration routes. In contrast, a 2024 updated study (39) suggested that delivery methods directly targeting the intestinal tract, such as endoscopic delivery, nasojejunal tube infusion, or rectal enema, yielded significantly better clinical outcomes than controls (*p <* 0.05), whereas the oral capsule approach conferred no therapeutic benefit.

Therefore, in addition to evaluating the overall antidepressant efficacy of FMT, this study synthesized evidence from high-quality RCTs and performed subgroup analyses based on administration route, follow-up duration, and underlying disease characteristics, thereby providing a more nuanced and targeted assessment of its potential role in mood regulation. We further appraise the certainty of evidence using the Grading of Recommendations Assessment, Development, and Evaluation (GRADE) framework, thereby providing a more targeted and methodologically transparent assessment of FMT’s clinical utility for depressive symptoms.

## 2 Methods

### 2.1 Study design

This study was conducted in alignment with the Preferred Reporting Items for Systematic Reviews and Meta-Analyses (PRISMA) guidelines for systematic reviews and meta-analyses (40). The protocol for this review was prospectively registered with the International Prospective Register of Systematic Reviews (PROSPERO) under the registration number CRD42025638185.

### 2.2 Literature search and selection

A comprehensive literature search was systematically conducted across five electronic databases: PubMed, Embase, Web of Science, the Cochrane Library, and CINAHL. The search covered publications from January 1, 2000, to December 31, 2024, as FMT techniques was not standardized in earlier literature, and did not receive broad scientific and clinical recognition until the early 2000s (41). The search strategy incorporated a combination of terms and their variants related to “Fecal Microbiota Transplantation” and “depression”. In addition to database searches, reference lists of all eligible publications were manually screened to identify potentially relevant studies not captured during the initial search. To ensure methodological rigor and consistency, two independent reviewers screened the titles, abstracts, and full texts of all identified studies. Any disagreements encountered during the selection process were resolved through consensus or by consultation with a third reviewer. The complete search algorithms are detailed in the Appendix 1.

Eligible studies included RCTs that evaluated FMT as a therapeutic intervention for depressive symptoms, regardless of dosage or route of administration. Comparators included placebo or autologous transplantation. Studies were required to report outcomes directly related to depressive symptoms and to be published in English. Exclusion criteria comprised non-human studies, non-clinical research, case reports, conference proceedings, narrative reviews, editorials, commentaries, systematic reviews, and meta-analyses.

### 2.3 Data extraction and quality assessment

To ensure data accuracy and methodological reliability, two reviewers independently extracted relevant information from the original publications. A standardized, pre-specified data extraction form was employed to systematically collect study characteristics, including general study details (e.g., first author and year of publication), participant demographics and sample sizes, FMT donor attributes, intervention protocols for both experimental and control groups, reported outcomes, and any adverse events. In cases where essential information was missing, efforts were made to contact the original study authors to retrieve the necessary data. Studies that failed to provide sufficient information were excluded from the final analysis.

The methodological quality of the included trials was independently appraised by two researchers using the Cochrane Risk of Bias assessment tool (42). This tool evaluates potential sources of bias across multiple domains, such as random sequence generation, blinding procedures, completeness of outcome data, selective outcome reporting, and other possible biases. Each domain was classified as having a “low,” “unclear,” or “high” risk of bias. Any discrepancies in assessment were resolved through discussion until consensus was reached.

### 2.4 Statistical analysis

Meta-analytical procedures were conducted using RevMan software version 5.4.1. Given the variability in measurement instruments across studies, the standardized mean difference (SMD) was calculated using Hedges’g. Statistical heterogeneity among the included studies was assessed using the I² statistic and the Chi-square test. In light of the clinical heterogeneity inherent in pooling distinct populations, random-effects models were employed as the primary analytic approach to provide more conservative and generalizable estimates. In cases of substantial heterogeneity (*p* ≤ 0.1 or I² ≥ 50%), additional subgroup analyses, sensitivity analyses, or narrative synthesis were conducted to explore sources of inconsistency. The overall effect size was evaluated using the Z statistic, and statistical significance was defined as *p* < 0.05. A summary of sensitivity analyses comparing model choices has been included in the Supplementary Materials to ensure robustness of findings.

### 2.5 Grading of the certainty of evidence

The certainty of evidence reflects the degree of confidence in the accuracy of estimated treatment effects derived from the available research. In this study, the strength of evidence across all reported outcomes was evaluated using the Grading of Recommendations Assessment, Development, and Evaluation (GRADE) framework (43). Following the guidelines outlined in the GRADE handbook, the overall certainty of evidence (CoE) was assessed by considering five key domains for potential downgrading: risk of bias, inconsistency, indirectness, imprecision, and publication bias. The CoE for each outcome was categorized as high, moderate, low, or very low, and the rating process was carried out using the GRADEpro online tool (accessible at http://www.gradepro.org).

## 3 Results

### 3.1 Search results and study characteristics

**Figure 1:**
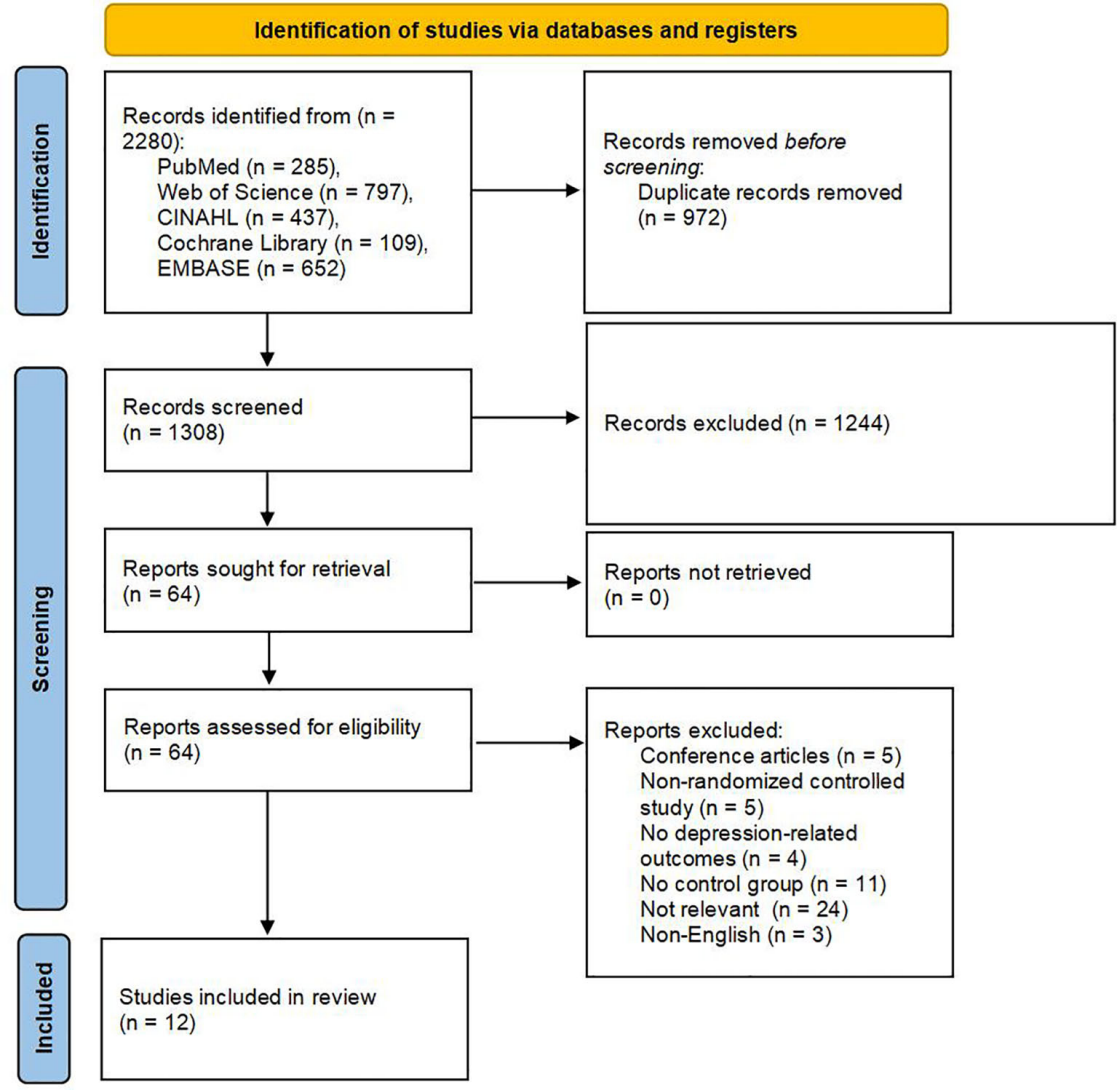
Study flow diagram.

An initial total of 2,280 records was identified through comprehensive searches across multiple electronic databases. After removing duplicates, 1,308 unique articles remained for further evaluation. Title and abstract screening resulted in the selection of 64 studies for full-text review, based on their potential relevance to the inclusion criteria. Of these, 51 articles were excluded following detailed assessment due to various reasons such as study design, population mismatch, or insufficient outcome reporting. Ultimately, 12 RCTs met the predefined eligibility criteria and were included in the final analysis (44–55). A detailed summary of the study selection process is illustrated in **Figure 1**.

The included studies were published between 2019 and 2024, representing diverse geographic regions such as China, Australia, Canada, Finland, and the United States. The sample sizes varied notably, ranging from 9 pairs to 136 pairs of participants, with a cumulative total of 347 participants in the FMT groups and 334 in the control groups. Across these studies, FMT was investigated as an intervention for various health conditions. Specifically, five trials examined disorders of gut-brain interaction (DGBIs), all of which focused on IBS (44, 52–55), two targeted neurological disorders (48, 49), another two focused on chronic illnesses accompanied by depressive symptoms (45, 53), and one study specifically evaluated patients diagnosed with major depressive disorder (47). However, all trials uniformly assessed the effect of FMT on depressive symptoms. Across studies, the mean age of participants ranged from 32.7 to 67.2 years, and the proportion of male participants varied between 13% and 87%.

FMT was delivered through various routes, including oral capsules (44, 45, 49, 52, 53, 55), colonoscopy (51, 54), gastroscopy (50), jejunal catheter (46), transendoscopic enteral tubing (TET) (48), and rectal enema (47). Controls included placebo capsules, autologous fecal microbiota transplantation, or standard medication treatment. Treatment frequencies ranged from a single administration to repeated cycles over several weeks or months. Donor screening was rigorous across all studies, though donor age and recruitment criteria varied. Most trials restricted the use of antibiotics, probiotics, and prebiotics during the intervention period; however, some studies failed to report the bowel preparation methods employed prior to FMT (47, 50, 52, 53, 55). Measurement of depressive symptoms was accomplished using various instruments: four studies applied the Hamilton Depression Rating Scale (HAM-D) (44, 48, 52, 53), two studies utilized the Hospital Anxiety and Depression Scale (HADS) (46, 55), and the remaining six studies each selected distinct scales, such as the Montgomery–Asberg Depression Rating Scale (MADRS) (45, 47, 51), the Cantonese version of the Geriatric Depression Scale (GDS-15) (49), Beck Depression Inventory (BDI) (54), and the Self-Rating Depression Scale (SDS) (50). Follow-up durations across these trials ranged widely, from a minimum of 2 weeks up to 12 months. A comprehensive overview of the included studies’ characteristics is detailed in **Table 1**.

**Table 1:** Study characteristics.

Study	Patients	Sample size (T/C)	Age M ± SD	%M	CoMedications	Bowel prep	Procedure of FMT	Comparator intervention	Donor selection	Study setting	Follow-up time	Depression assessment tools
Zhang et al., 2024 China (44)	IBS	P: 8 R: 10 B: 9	P: 40.8 ± 8.3 R: 41.6 ± 11.8 B: 35.2 ± 12.8	P: 87% R: 80% B: 56%	Prohibited during study: antibiotics, probiotics, prebiotics.	6h fasting	P: Donor-recipient-matched FMT capsules. R: Random-donor FMT capsules. Dose: 40 capsules each were taken on days 1, 3 and 5 of the study. Route: Oral.	Placebo capsules.	Donor-recipient-matched and Random-donor.	Hospital outpatient	Baseline, weeks 4, 8, and 12	HADS
Jiang et al., 2024 China (45)	COVID-19 with associated diarrhea and depressive symptoms	19/20	T: 32.7 ± 7.3 C: 37.6 ± 7.6	T: 37% C: 45%	Prohibited during study: Antibiotics, probiotics.	8h fasting	Capsules encapsulated fecal microbiota. Dose: 40 capsules. 10 capsules/day for 4 days. Route: Oral.	Identical capsules without microbiota.	Three healthy donors (two men, one women) who were selected following a rigorous screening protocol.	Hospital outpatient	Baseline, Day 7	Depression severity
Fang et al., 2024 China (46)	Fibromyalgia	22/23	T: 52.8 ± 4.6 C: 55.3 ± 6.6	T: 18% C: 13%	Oral duloxetine 30 mg/bid.	Standard colonoscopy preparation.	Bacterial fluid (3-5×10¹¹ CFU/mL), combined with duloxetine therapy. Dose:​250mL per dose, 3 consecutive days. Route: Jejunostomy catheter.	Duloxetine therapy alone.	Two healthy adults (18–25 years old), screened for infections, chronic diseases, and drug resistance.	Pain clinic	Baseline, 1 week, 1, 2, 3, 6, and 12 months	HADS
Green et al., 2023 Australia (47)	MDD	10/5	T: 47.2 ± 6.5 C: 38.4 ± 2.1	T: 40% C: 20%	Stable treatment pharmacotherapy	NR	Dose:​4×50mL (total 50g stool) Route:​Enema	Identical enemas without stool content.	Donors screened for infections and gastrointestinal health.	Hospital research unit	Baseline, Week 2, Week 8	MADRS
Tian et al., 2023 China (48)	PSP-RS	34/34	T: 67.1 ± 5.1 C: 67.2 ± 5.1	T: 62% C: 56%	Oral ciprofloxacin and metronidazole for 5 days before transplantation.	Standard colonoscopy preparation.	Dose:​200mL fecal suspension ×7 days/cycle, total 3 cycles. Route:​Transendoscopic enteral tubing (TET)	Saline and food coloring via the same route.	16 healthy college students were selected through questionnaire, medical interview, and laboratory tests.	University tertiary referral hospital	Baseline, weeks 2, 7, 12, 16, and 36	HAMD
Cheng et al., 2023 China (49)	PD with gastrointestinal disorders	27/27	T: 60.5 ± 8.7 C: 62.6 ± 8.4	T: 56% C: 63%	Oral Levodopa or Dopamine agonists Prohibited during study: Antibiotics	Fasting	Oral FMT capsules. Dose: 16 capsules (≈50g donor stool), once a week for 3 weeks. Route: Oral.	Placebo capsules containing saline and food coloring.	Four healthy donors screened through questionnaires, medical history, and laboratory tests.	Hospital Department of Neurology	Baseline, weeks 4, 8, and 12	GDS-15
Shang et al., 2023 China (50)	UC	136/136	T: 45.2 ± 13.6 C: 40.6 ± 12.9	T: 50% C: 50%	Discontinued mesalamine before FMT.	NR	Bacterial suspension. Dose: 150ml (from 50g donor stool), 3 treatments, once every 3 weeks. Route: Gastroscope to descending duodenum.	Equal volume saline via identical procedure.	136 healthy adolescents (68 males and 68 females) aged 10–18 years.	Hospital	Baseline, 3, 6, and 9 weeks	SDS
Ghorbani et al., 2023 Canada (51)	Severe obesity and insulin resistance	15/13	T: 45.0 ± 5.6 C: 48.0 ± 7.1	T: 20% C: 23%	Excluded probiotics/prebiotics/antibiotics within 3 months.	Standard colonoscopy preparation	Allogenic FMT (donor stool). Dose: Single dose (filtrate from 50g stool). Route: Colonoscopy.	Autologous FMT (own stool).	Three screened healthy donors (2 male, 1 female; mean age 31.7 years).	Hospital	Baseline, 1, 3 months	MADRS
Guo et al., 2021 China (52)	IBS combined with mild to moderate anxiety and depression	9/9	T: 44.0 ± 4.3 C: 50.0 ± 5.7	T: 56% C: 56%	Excluded probiotics/prebiotics/antibiotics within 2 weeks.	NR	Oral FMT capsules. Dose: 30 capsules/dose, 3 doses total (every 2 days). Route: Oral capsules.	Identical oral empty capsules.	Healthy donors screened through questionnaires, medical history, and laboratory tests.	Hospital	1, 8, and 12 weeks	HAM-D
Lin et al., 2021 China (53)	IBS combined with mild to moderate anxiety and depression	9/9	T: 44.3 ± 9.3 C: 50.4 ± 10.7	T: 56% C: 56%	Excluded probiotics/prebiotics/antibiotics within 2 weeks.	NR	Oral FMT capsules. Dose: 30 capsules/dose, 3 doses total (every 2 days). Route: Oral capsules.	Identical oral empty capsules.	Screened according to FMT donor guidelines (2019).	Hospital	Baseline, 1, 2, and 3 months.	HAM-D
Lahtinen et al., 2020 Finland (54)	IBS	23/26	T: 47.3 ± 16.8 C: 46.3 ± 14.3	T: 48% C: 35%	Excluded antibiotic/probiotic use within 2 weeks pre-study.	Standard colonoscopy preparation.	Dose: 30g fecal suspension. Route: Single colonoscopy administration.	Autologous fecal transplant (identical preparation/administration)	One universal donor screened for infectious diseases, normal health profile.	Multiple Hospitals	4, 8, 12, 26, and 52 weeks.	BDI
Aroniadis et al., 2019 USA (55)	IBS	25/23	T: 33.0 ± 4.8 C: 42.0 ± 3.9	T: 64% C: 61%	Excluded probiotics/prebiotics/antibiotics within 2 weeks.	NR	Dose: 75 capsules over 3 days (25/day). Route: Oral capsules.	Identical capsules without stool content.	Four healthy donors screened for infections.	Medical Center and the Medical Research Center	Baseline, 12 weeks.	HADS

IBS, Irritable Bowel Syndrome; MDD, Major Depressive Disorder; PSP-RS, Progressive Supranuclear Palsy-Richardson’s Syndrome; PD, Parkinson’s Disease; UC, Ulcerative Colitis; T, Treatment groups; C, Control groups; P, Donor-recipient matched FMT group; R, Random-donor FMT group; B, Placebo group; %M=Proportion of male; NR, Not reported; HADS, Hospital Anxiety and Depression Scale; MADRS, Montgomery-Åsberg Depression Rating Scale; HAMD, Hamilton Depression Scale; GDS-15, Geriatric Depression Scale-15; SDS, Self-Evaluation of Depression; BDI, Beck Depression Inventory.

### 3.2 Study quality

**Figure 2:**
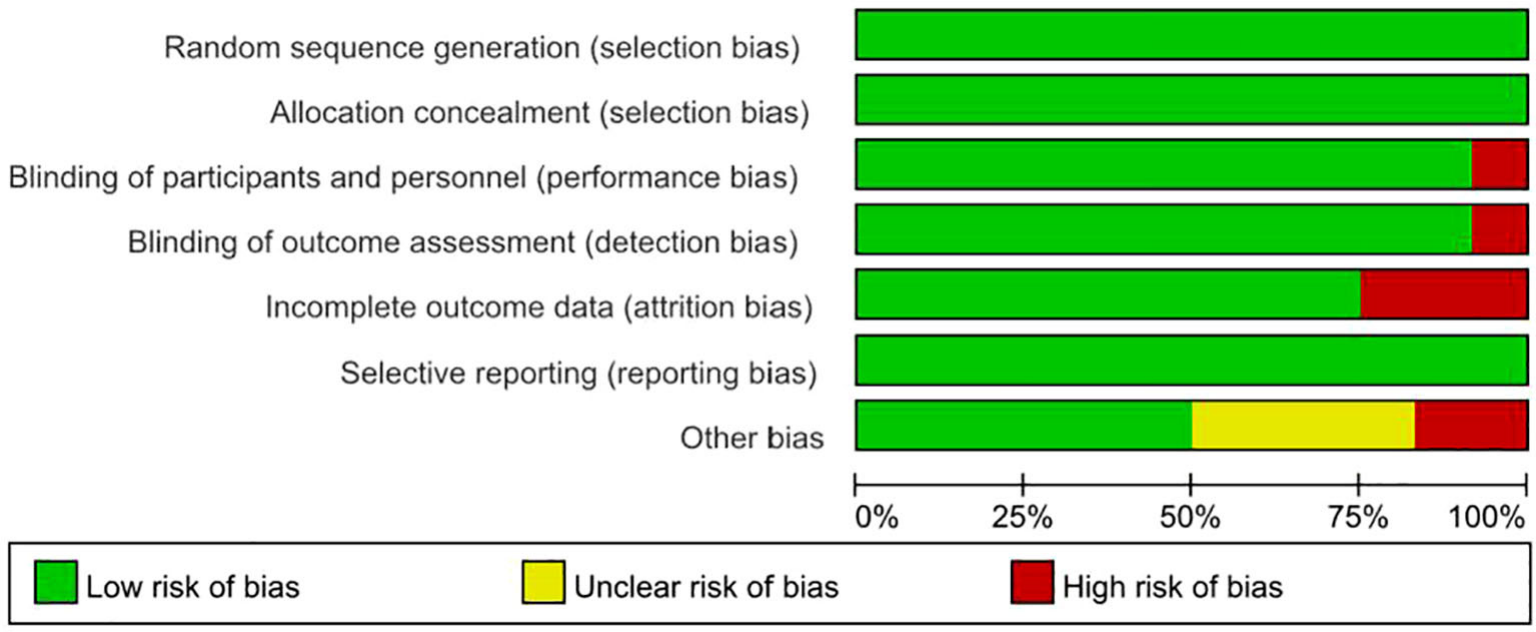
Risk of bias graph: review authors’ judgements about each risk of bias item presented as percentages across all included studies.

**Figure 3:**
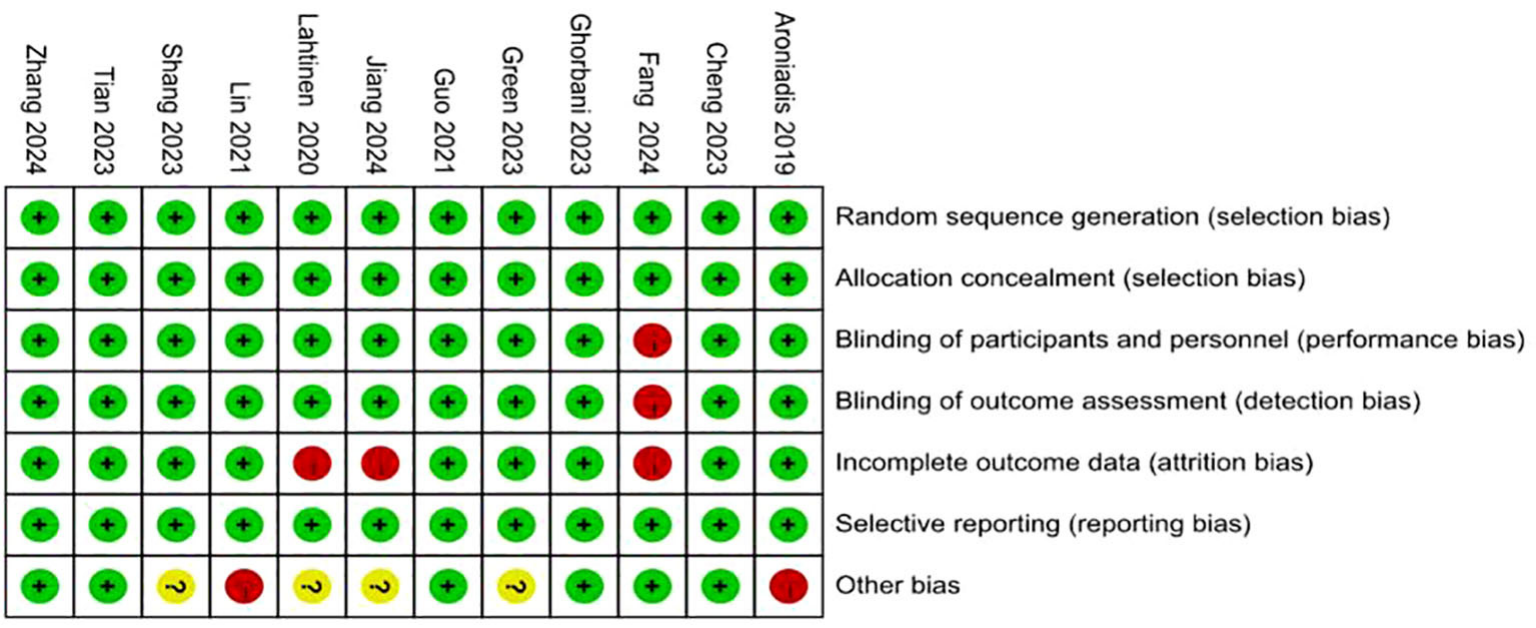
Risk of bias summary: review authors’ judgements about each risk of bias item for each included study.

Risk of bias was assessed in accordance with the guidelines outlined in the Cochrane Handbook for Systematic Reviews of Interventions. The detailed evaluations are presented in **Figures 2** and **3**. All included studies provided explicit descriptions of their randomization procedures, with two trials (54, 55) employing block randomization techniques. The trial conducted by Fang et al. (46) was an open-label study, and thus did not implement allocation concealment or blinding procedures.

In contrast, the remaining studies incorporated appropriate methods for allocation concealment, utilized placebo-controlled designs, and ensured blinding of participants, intervention providers, and outcome assessors. Two studies (46, 54) documented participant withdrawal following randomization and provided reasons for attrition; however, they did not specify how missing data were addressed. One study did not clearly define the depression outcome, and no validated measurement tool was specified (45). The rest of the trials were judged to have a low risk of bias with respect to incomplete outcome data and selective reporting. Two studies reported insufficient sample sizes (53) and author conflicts of interest (55), introducing a potential source of bias.

### 3.3 Publication bias

**Figure 4:**
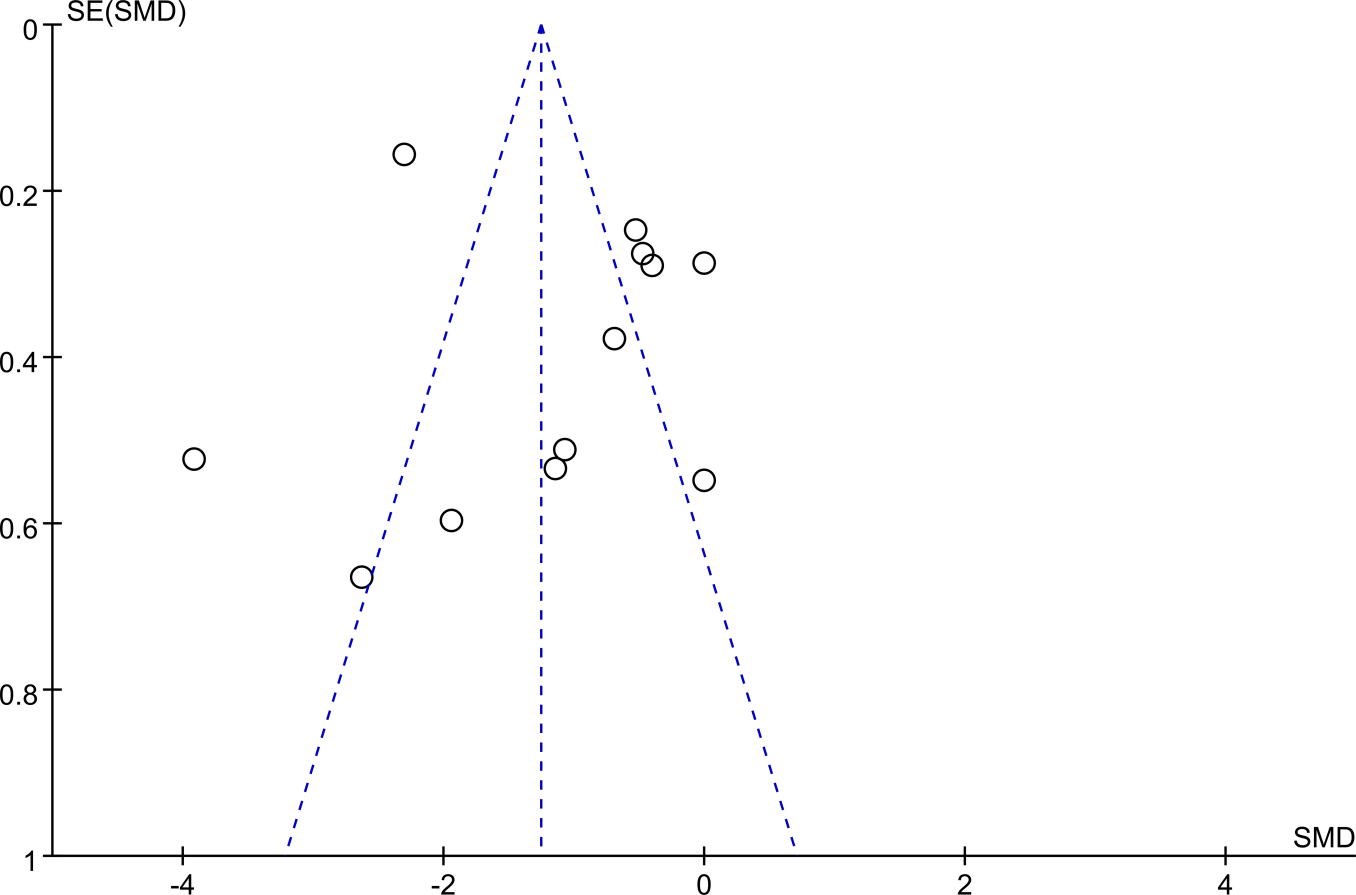
Funnel Plot.

Publication bias was assessed through visual inspection of a funnel plot. The distribution of studies appeared to be approximately symmetrical, suggesting no substantial indication of publication bias. A detailed depiction of the funnel plot is provided in **Figure 4**.

### 3.4 Meta-analysis results

#### 3.4.1 Overall effect of FMT on depressive symptoms

**Figure 5:**
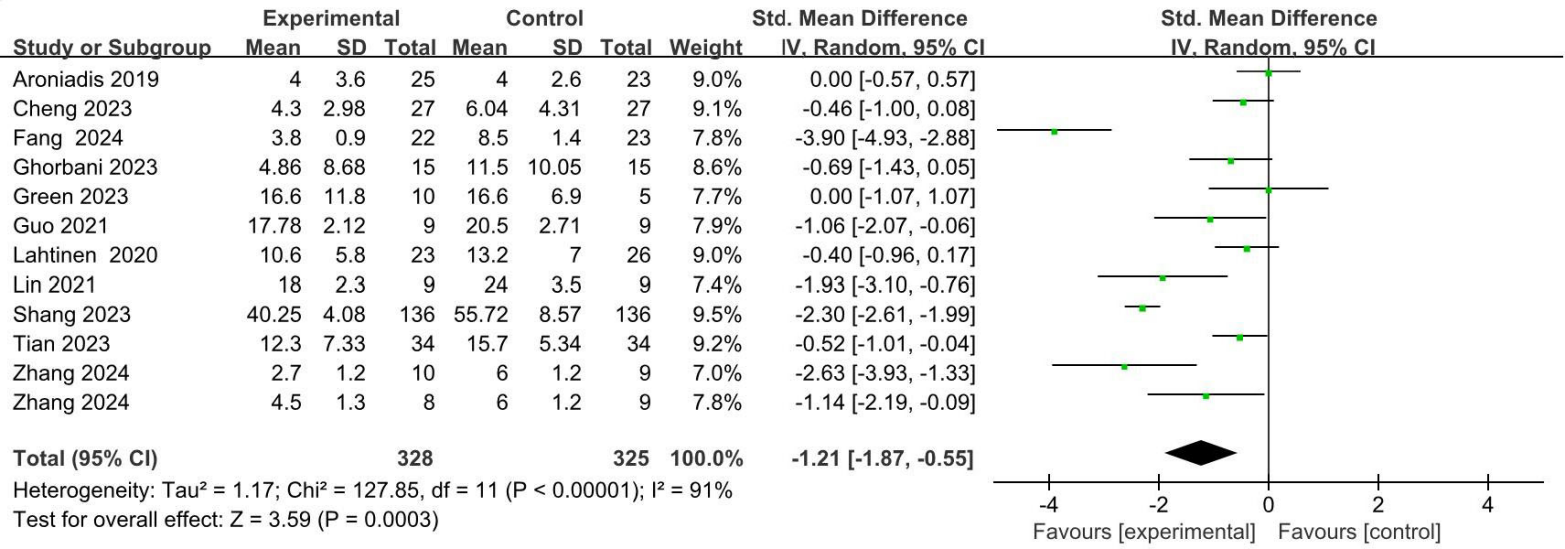
Overall effect of FMT on depressive symptoms.

A total of 11 studies (44, 46–55), involving 644 patients, provided quantitative data relevant to depressive symptoms. In the trial conducted by Zhang et al. (44), participants receiving FMT were categorized into two distinct intervention groups based on donor-recipient matching: the donor-recipient matched group and the randomized donor group; both groups were subsequently included in the meta-analysis. In cases involving multiple follow-up assessments, only data from the final follow-up were utilized as the outcome measure. Due to variations in depression assessment instruments across the included studies, a random-effects model was applied to estimate the SMD between the intervention and control groups. The pooled results indicated a significant reduction in depressive symptoms in the FMT groups compared to the controls (SMD = -1.21; 95% CI: -1.87 to -0.55; *p* = 0.0003) (**Figure 5**). Nevertheless, considerable heterogeneity was observed among the studies (I² = 91%, *p* < 0.0001). Jiang et al. (45) employed a generalized linear mixed model (GLMM) to examine the effect of FMT on depressive symptoms in patients with mild to moderate COVID-19. Their analysis demonstrated a significant decrease in depression scores among individuals in the FMT group (β = -1.046; *p* = 0.006), with an accelerating therapeutic effect observed over time, as evidenced by the significant interaction between treatment and time (*p* = 0.009).

Sensitivity analysis was subsequently performed by excluding three studies: Fang et al. (46), Shang et al. (50), and the donor-recipient matched subgroup from Zhang et al. (44). After excluding these studies, the analysis of the remaining nine RCTs, including the randomized donor subgroup from Zhang et al. (44), showed a notable reduction in heterogeneity (I² = 37%). Furthermore, the depressive symptom scores in the FMT group remained significantly improved compared to the controls (SMD = -0.56; 95% CI: -0.86 to -0.26; *p* = 0.0003) (Appendix 2: **Supplementary Figure S1**). These findings suggest that the excluded studies may substantially contribute to the observed overall heterogeneity, potentially due to marked differences in sample size or distinct intervention methodologies. To further explore the possible sources of heterogeneity, subgroup analyses were conducted based on the routes of FMT administration, duration of follow-up, and the clinical characteristics of the participants.

#### 3.4.2 Effect of different FMT delivery routes on depressive symptoms

**Figure 6:**
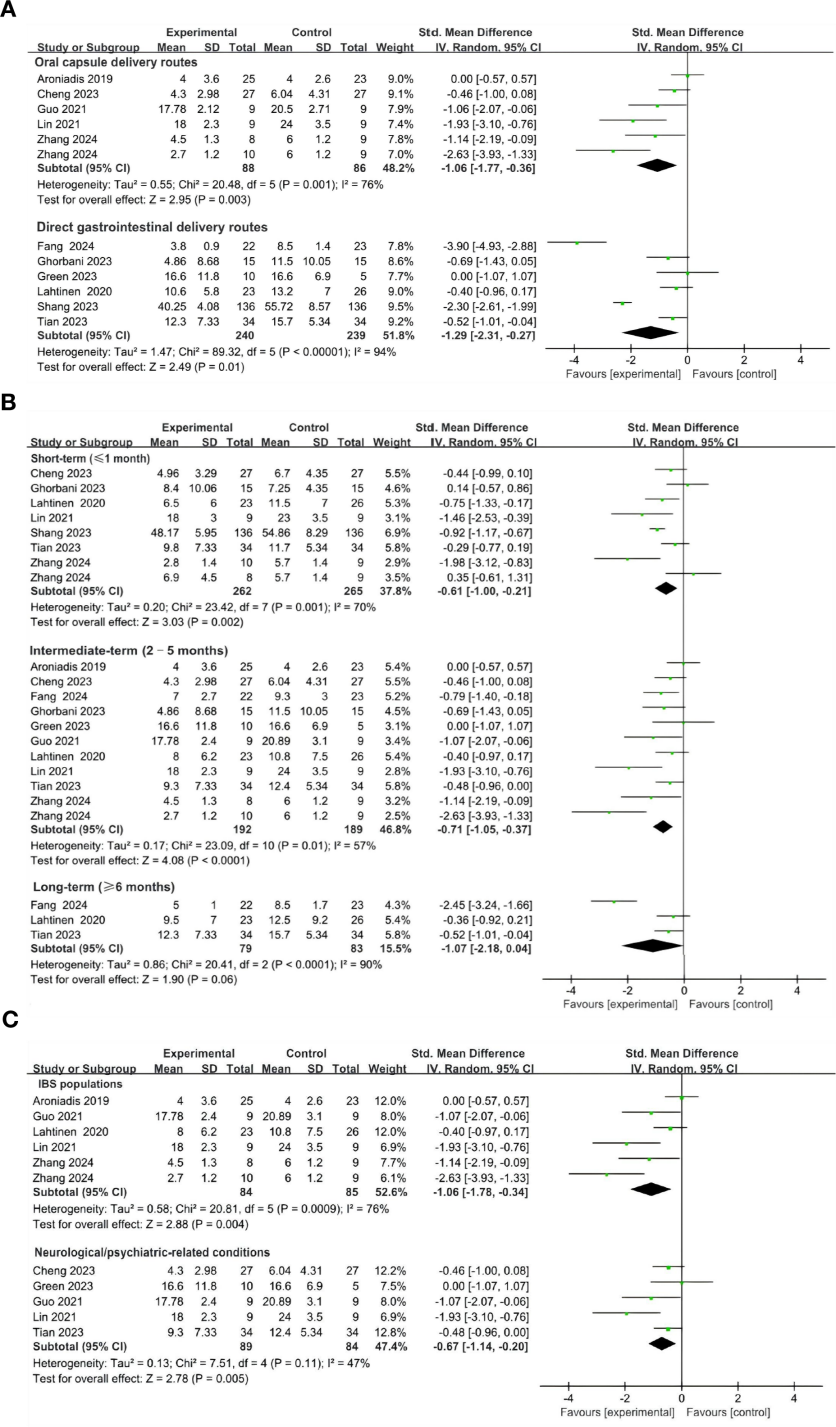
Subgroup analysis of the effects of FMT on depressive symptoms. **(A)** Effect of different FMT delivery routes on depressive symptoms. **(B)** Effect of FMT on depressive symptoms at different follow-up time. **(C)** Effects of FMT on depressive symptoms across different clinical populations.

The meta-analysis indicated that FMT administered via oral capsules (44, 49, 52, 53, 55), resulted in a statistically significant alleviation of depressive symptoms compared with control groups (SMD = -1.06; 95% CI, -1.77 to -0.36; *p *= 0.0003) (**Figure 6A**). Similarly, direct gastrointestinal delivery routes—including colonoscopy (51, 54), jejunostomy catheter (46), transendoscopic enteral tubing (TET) (48), gastroscopy (50), and rectal enema (47) —also demonstrated significant improvements in depressive outcomes (SMD = -1.29; 95% CI, -2.31 to -0.27; *p *= 0.01) (**Figure 6A**).

To evaluate the potential heterogeneity among direct gastrointestinal FMT delivery routes, we conducted a series of sensitivity analyses. Two studies involving direct upper gastrointestinal administration of FMT were excluded (46, 50). The pooled effect sizes remained statistically significant (SMD = -0.47; 95% CI, -0.78 to -0.15; *p *= 0.004; I^2^ = 0), indicating the robustness of FMT’s therapeutic effect on depressive symptoms across vdirect gastrointestinal delivery approaches (Appendix 2: **Supplementary Figure S2**).

#### 3.4.3 Effect of FMT on depressive symptoms at different follow-up time

To evaluate the sustained therapeutic effects of FMT on depressive symptoms, we performed a subgroup analysis based on follow-up duration, categorizing studies into short-term (≤1 month) (44, 48–51, 53, 54), intermediate-term (2–5 months) (44, 46–49, 51–55), and long-term (≥ 6 months) (46, 48, 54)follow-up periods. Significant improvements in depressive symptoms were observed during the short-term (SMD = -0.61; 95% CI, -1.00 to -0.21; *p* = 0.002) (**Figure 6B**). In the intermediate-term periods post-treatment, the effectiveness of FMT was even more pronounced (SMD = -0.71; 95% CI, -1.05 to -0.37; *p* < 0.0001) (**Figure 6B**), while the effect was attenuated in the longer-term group (95% CI, -2.18 to 0.04; *p* = 0.06) (**Figure 6B**). These findings suggest that FMT provides significant symptomatic relief in depressive states during short- to medium-term follow-up periods; however, further investigations are necessary to clarify the durability of its long-term effects.

#### 3.4.4 Effects of FMT on depressive symptoms across different clinical populations

In the subgroup of participants with DGBIs—IBS (44, 52–55), FMT significantly reduced depressive symptoms compared with controls (SMD = -1.06, 95% CI: -1.78 to -0.34, p = 0.004), although heterogeneity was substantial (I² = 76%) (**Figure 6C**). Five RCTs enrolling participants with neurological/psychiatric-related conditions, including Parkinson’s disease (PD) (49), progressive supranuclear palsy–Richardson’s syndrome (PSP-RS) (48), major depressive disorder (MDD) (47), and IBS with comorbid depression (52, 53), also demonstrated a significant benefit of FMT over controls (SMD = -0.67, 95% CI: -1.14 to -0.20, *p* = 0.005; I² = 47%) (**Figure 6C**).

### 3.5 Certainty of evidence

The overall certainty of evidence ranged from low to very low across the included outcomes. A comprehensive summary of the quality ratings is provided in **Table 2**.

**Table 2:** GRADE summary of findings for all outcomes.

Quality assessment							No of patients		Effect	Quality
No of studies	Design	Risk of bias	Inconsistency	Indirectness	Imprecision	Other considerations	FTM	Control		
Overall Effect of FMT on Depressive Symptoms (Better indicated by lower values)										
11	RCTs	serious^1^	serious^2^	serious^3^	no serious imprecision	none	328	325	SMD 1.21 lower (1.87 to 0.55 lower)	ⴲⵔⵔⵔ VERY LOW
Subgroup by different FMT delivery routes - Oral capsule delivery routes (Better indicated by lower values)										
5	RCTs	no serious risk of bias	serious^2^	serious^3^	serious^4,5^	none	88	86	SMD 1.06 lower (1.77 to 0.36 lower)	ⴲⵔⵔⵔ VERY LOW
Subgroup by different FMT delivery routes - Direct gastrointestinal delivery routes (Better indicated by lower values)										
6	RCTs	serious^1^	serious^2^	serious^3^	no serious imprecision	none	240	239	SMD 1.29 lower (2.31 to 0.27 lower)	ⴲⵔⵔⵔ VERY LOW
Subgroup by different follow-up time - Short-term (≤1 month) (Better indicated by lower values)										
7	RCTs	no serious risk of bias	serious^2^	serious^3^	no serious imprecision	none	262	265	SMD 0.61 lower (1 to 0.21 lower)	ⴲⵔⵔⵔ LOW
Subgroup by different follow-up time - Intermediate-term (2–5 months) (Better indicated by lower values)										
10	RCTs	serious^1^	no serious inconsistency	serious^3^	serious^4,5^	none	192	189	SMD 0.71 lower (1.05 to 0.37 lower)	ⴲⵔⵔⵔ VERY LOW
Subgroup by different follow-up time - Long-term (≥6 months) (Better indicated by lower values)										
3	RCTs	serious^1^	serious^2^	serious^3^	serious^4,5^	none	79	83	SMD 1.07 lower (2.18 lower to 0.04 higher)	ⴲⵔⵔⵔ VERY LOW
Subgroup by different disease characteristics - IBS populations (Better indicated by lower values)										
5	RCTs	no serious risk of bias	serious^2^	no serious indirectness	serious^4,5^	none	84	85	SMD 1.06 lower (1.78 to 0.34 lower)	ⴲⵔⵔⵔ LOW
Subgroup by different disease characteristics - Neurological/psychiatric-related conditions (Better indicated by lower values)										
5	RCTs	no serious risk of bias	no serious inconsistency	serious^3^	serious^5^	none	89	84	SMD 0.67 lower (1.14 to 0.2 lower)	ⴲⵔⵔⵔ LOW

^1^ Some studies have a high risk of bias.

^2^ I^2^ > 60%.

^3^ The subjects had different diseases.

^4^ The sample size was insufficient.

^5^ Some studies use different depression assessment tools.

## 4 Discussion

### 4.1 Overall effect of FMT on depressive symptoms

FMT has demonstrated potential therapeutic benefits in the treatment of various psychiatric and psychological disorders (56, 57). In this meta-analysis of 12 RCTs involving 681 participants, FMT significantly alleviated depressive symptoms compared with placebo or standard pharmacological treatment, with no evidence of publication bias. Although sensitivity analyses revealed a reduction in the absolute effect size following the exclusion of studies with large sample sizes or atypical designs (from SMD = -1.21 to SMD = -0.56), the overall statistical significance remained robust (*p *= 0.0003). This suggests that the therapeutic impact of FMT on depressive symptoms is stable and may be effective even in studies involving smaller cohorts. Of particular interest, the findings of Jiang et al. (45) reinforce the notion that FMT exerts a sustained and progressively enhanced antidepressant effect, which is consistent with the overall trends observed in the present meta-analysis. However, this study did not provide a clear definition of the depression outcome, nor did it specify the use of a validated measurement tool.

The therapeutic benefit of FMT may be explained by its ability to correct dysbiosis, restore gut microbial diversity, and modulate the MGBA, thereby influencing neuroinflammatory processes, neurotransmitter metabolism, and host immune responses (21, 35, 58). Specifically, the enrichment of anti-inflammatory taxa and restoration of SCFA-producing bacteria following FMT could attenuate systemic inflammation and improve serotonergic signaling—both of which are implicated in the pathophysiology of depression.

Notably, the heterogeneity across included trials was substantial (I² = 91%), indicating considerable variability in observed effect sizes. This high heterogeneity is likely multifactorial, arising from differences in study populations, variability in FMT delivery routes follow-up durations, and the use of diverse depression assessment tools with differing sensitivity and specificity. Furthermore, differences in FMT preparation protocols (fresh vs. frozen stool, single vs. repeated administration), donor selection criteria, and particularly donor sources (single donor vs. pooled donors, related vs. unrelated donors) may substantially influence microbiota composition and functional capacity, thereby contributing to outcome variability (59). In addition, variations in pre-treatment and baseline microbiota assessment further complicate interpretation. For example, Tian et al. (48) reported that recipients received oral ciprofloxacin and metronidazole for five days prior to FMT, whereas other studies only restricted antibiotic use during the intervention period. Zhang et al. (44) evaluated both donor and recipient microbiota, and in the matched FMT group, donor–recipient matching was based on gut microbiota structure (40.3%), diversity (23.2%), beneficial bacteria (25.2%), and harmful bacteria (11.3%). Seven additional trials (45, 46, 48, 50, 51, 54, 55) also assessed baseline microbiota, but none systematically analyzed whether recipients’ baseline microbial composition significantly influenced FMT success or engraftment. Recent evidence underscores the importance of this issue. Porcari et al. (60) demonstrated that higher recipient microbial diversity and greater compatibility between donor and recipient microbiota were key determinants of donor strain engraftment and clinical response. None of the included trials systematically evaluated these factors, which may partly explain the variability in FMT effectiveness observed across studies. While random-effects modeling accounts for some of this variability, the interpretation of pooled estimates should be made cautiously.

### 4.2 Effect of different FMT delivery routes on depressive symptoms

In the present study, both oral capsule and direct gastrointestinal FMT delivery routes produced significant improvements in depressive symptoms. Nevertheless, differences were evident between these routes, with the direct gastrointestinal group exhibiting a larger effect size in alleviating depressive symptoms (SMD: −1.29 vs −1.06). This disparity might stem from variations in microbial colonization efficiency, the pace of gut microbiota reconstitution, and patient compliance associated with each method (36, 61, 62).

Directly delivering donor microbiota to the colonic environment via endoscopy or enema likely facilitates colonization and functional activity more effectively, possibly because the colon is the primary site for microbiota colonization (63, 64). Studies have shown that after FMT administered via gastroscopy in IBS patients, the recipients’ gut microbiota profiles shift significantly toward those of the donor, a change strongly associated with symptom improvement (65, 66). Conversely, the oral capsule route may expose the introduced microbes to gastric acid and the heterogeneous gastrointestinal environment, thereby reducing the viability and functional stability of the transplanted bacteria (67). In line with these mechanistic insights, clinical data have confirmed that the endoscopic administration group achieves superior neurotransmitter modulation: after treatment, serotonin (5-HT) and γ-aminobutyric acid (GABA) levels are significantly elevated while glutamate levels are reduced, and the magnitude of these neurochemical changes far exceeds that observed with other delivery methods (46). Furthermore, the mechanical stimulation involved in endoscopic procedures might enhance signaling along the gut–brain axis and promote vagus nerve-mediated anti-inflammatory pathway activation, thereby amplifying the modulation of depression-related inflammatory factors (35, 68). On the other hand, oral capsule FMT is more convenient and safer than enema or endoscopic approaches, and its high patient compliance makes it suitable for long-term maintenance therapy (69). With repeated administration, FMT can facilitate the gradual restoration of the recipient’s gut microbiota diversity and stability, thereby extending the duration of disease remission. Therefore, the selection of an FMT delivery route should comprehensively consider factors such as microbial viability, colonization efficiency, patient compliance, procedural invasiveness, and the feasibility of repeated administration.

However, it is important to acknowledge that this methodological subgroup analysis focused solely on the delivery route, without accounting for several other potentially influential methodological factors. These include heterogeneity in donor selection protocols, variation in stool dosage and frequency, differences in bowel preparation procedures (e.g., fasting, colonoscopy prep, antibiotic preconditioning), and concomitant use of antibiotics, probiotics, or psychotropic medications. Such factors may act as confounders and contribute to variability in FMT outcomes. Therefore, while our findings suggest a potential advantage of direct gastrointestinal delivery, these results should be interpreted with caution, and future studies are warranted to isolate and systematically assess the influence of individual methodological components on clinical efficacy.

### 4.3 Effect of FMT on depressive symptoms at different follow-up time

Existing research indicates that FMT exerts its therapeutic effects through mechanisms such as modulating gut microbiota composition, restoring microbiota-gut-brain axis function, ameliorating neuroinflammation, and rectifying neurotransmitter imbalances (35). However, therapeutic outcomes have been observed to vary at different follow-up time points.

Clinical observations suggest that FMT can improve psychiatric symptoms in the short term. For instance, one study focusing on patients with functional constipation accompanied by psychiatric symptoms found that within four weeks after FMT, patients experienced significant relief in both gastrointestinal and psychiatric symptoms (70). Similarly, in patients with chronic insomnia, FMT significantly improved insomnia symptoms at the 4-week mark and also had a positive effect on co-occurring anxiety and depression (30). Animal experiments further corroborate these clinical findings. In a mouse model of depression, FMT rapidly reversed depressive-like behavior, with especially pronounced effects in mice that were unresponsive to SSRIs (71). Another study demonstrated that transplanting fecal microbiota from volunteers experiencing psychological stress and subclinical depressive symptoms into mice induced depression- and anxiety-like behaviors within a short period (72), providing inverse evidence for the gut microbiota’s rapid influence on psychiatric symptoms.

Mid-term follow-up studies have revealed that FMT’s effects can be both persistent and stable. For example, a study in patients with treatment-resistant depression reported that at 12 weeks post-FMT, depression scores were significantly reduced, and this improvement was associated with stable changes in gut microbiota composition (73). Notably, improvements in psychiatric symptoms with FMT often coincide with relief of gastrointestinal symptoms. In patients with IBS, FMT not only alleviated gastrointestinal symptoms but at mid-term follow-up it also exerted a positive effect on mood (74). This dual “gut-brain” improvement effect reinforces the crucial role of the microbiota-gut-brain axis in psychiatric disorders (75).

Research on FMT’s long-term effects is relatively limited, and available data suggest that the therapeutic outcomes may vary between individuals. Some studies have reported that improvements in depressive symptoms are sustained at six months post-FMT (76), whereas others have noted a gradual diminishment of efficacy (47, 77).

In our study, we found that while FMT significantly alleviated depressive symptoms at short- and mid-term follow-ups, its effect had waned by the long-term follow-up and was no longer statistically significant. This divergence could be attributable to several factors. First, the ecological stability of the gut microbiome may be insufficient; the exogenous microbial strains introduced via FMT might not be able to colonize the host gut in the long term, causing the microbiota composition to gradually revert to its pre-transplant state (35). Second, donor-specific microbiota–induced changes in the host serum metabolome may diminish as metabolic homeostasis is restored (46, 73, 78). Finally, current clinical practice typically employs only a single FMT session, whereas studies suggest that repeated FMT can enhance efficacy by continuously modulating gastrointestinal symptoms and maintaining gut microbial diversity. This implies that the attenuation of long-term effects may be related to an insufficient frequency of intervention (79). Additionally, the propensity for depressive symptoms to relapse could offset FMT’s initial benefits. Research indicates that even if FMT achieves symptom remission, patients may later experience a return of gut dysbiosis and neuroinflammatory activation due to environmental stressors or genetic predispositions, potentially precipitating a relapse of depression (58, 80). In summary, sustaining the long-term efficacy of FMT will likely require further optimization of microbiota transplantation strategies and exploration of combined approaches to consolidate its therapeutic effects, such as adjunctive neuromodulation or lifestyle interventions.

### 4.4 Effects of FMT on depressive symptoms across different clinical populations

This study analysis demonstrated that FMT significantly improved depressive symptoms in both IBS populations and neurological/psychiatric-related conditions. Notably, the effect was numerically greater in IBS populations than in neurological/psychiatric-related conditions (SMD: −1.06 vs −0.67). Given that IBS is now classified as a disorder of DGBI (81, 82), these findings are particularly relevant. Survey data indicate that depressive symptoms in patients with DGBIs are closely associated with visceral hypersensitivity, suggesting that dysregulation of the MGB axis may be a core mechanism underlying this comorbidity (83). Gut dysbiosis may enhance FMT’s antidepressant efficacy by concurrently altering SCFA/5-HTP metabolism and exacerbating neuroinflammation.

Multiple factors could underlie this observation. First, the bidirectional regulation of the gut-brain axis is considered pivotal: FMT can modulate the gut microbiota composition or function, reduce systemic inflammation, and promote neurotransmitter synthesis, thereby concurrently improving gastrointestinal dysfunction and mood symptoms (35, 84). Moreover, individuals with IBS often exhibit compromised gut barrier function and dysbiosis. These pathological conditions can exacerbate depressive symptoms by activating vagal nerve pathways and immune-inflammatory responses (85, 86). FMT’s targeted restoration of the gut microenvironment in such cases may lead to more pronounced benefits for this subgroup (87). In addition, the potential additive effects of psychological interventions warrant attention. Studies have shown that interventions such as cognitive-behavioral therapy not only directly alleviate depressive mood but also relieve gastrointestinal symptoms by reducing visceral hypersensitivity and improving autonomic nervous regulation (88, 89). This dual mechanism may yield a synergistic effect in patients with gastrointestinal disorders. Furthermore, genetic research has revealed that depression and certain gastrointestinal diseases (e.g., IBS) share genetic loci and pleiotropic genes (90). This suggests that FMT might produce broader improvements by targeting common biological pathways. It should also be noted that even individuals who do not meet clinical diagnostic criteria for depression often exhibit subclinical depressive symptoms associated with chronic low-grade inflammation (91). Thus, FMT’s anti-inflammatory properties could play a regulatory role in such individuals as well.

Our findings contrast with those of Wang et al. (2021) (36), who reported non-significant effects of FMT on depressive symptoms when analyzed as a secondary outcome in patients with IBS. Specifically, their meta-analysis yielded no significant changes at 12 weeks (MD = −0.26, 95% CI −3.09 to 2.58), 24 weeks (MD = −2.26, 95% CI −12.96 to 8.45). Several methodological and clinical differences may explain these discrepant conclusions. First, Wang et al. employed MD values, which limit comparability across different depression rating scales, whereas our study used SMD to harmonize results across diverse tools (HAM-D, MADRS, HADS, SDS, BDI, GDS-15). Second, our analysis incorporated recently published RCTs reporting more robust antidepressant effects (44, 52), which were not included in Wang et al.’s synthesis. Collectively, these refinements may explain why our meta-analysis demonstrated significant improvements in depressive symptoms for both IBS populations and neurological/psychiatric-related conditions, thereby extending and updating the evidence base regarding FMT’s antidepressant potential.

Nonetheless, the substantial heterogeneity warrants cautious interpretation. In our study, all included studies involved the gut-brain axis and could manifest with depressive symptoms, only one trial formally assessed for MDD at baseline (47). Most trials evaluated depression as a secondary outcome or enrolled participants with subclinical depressive symptoms, in some cases with baseline depression scores already below the threshold for clinical significance. Therefore, the clinical relevance of improvements in depression scores remains uncertain. In these populations, such improvements may partly reflect amelioration of the underlying condition (e.g., IBS or neurological disorders) rather than a direct antidepressant effect of FMT.

### 4.5 Strengths and limitations of the study

This meta-analysis systematically evaluated the therapeutic efficacy of FMT on depressive symptoms across multiple patient populations, providing robust evidence of its broad clinical benefit. A major strength lies in the inclusion of diverse participant groups, including both patients formally diagnosed with depression and those without a clinical diagnosis but exhibiting depressive symptoms, thereby enhancing the generalizability of the findings. Additionally, subgroup analyses conducted by route of administration, disease characteristics and follow-up durations allowed for a nuanced understanding of factors affecting treatment efficacy. Finally, by highlighting the bidirectional interactions between the gut microbiota and brain function, our findings support the microbiota-gut-brain axis as a promising target for depression management, and provide a foundation for future research on personalized microbiota interventions.

This meta-analysis has several important limitations that should be considered when interpreting the findings. First, there was substantial heterogeneity in clinical populations, underlying conditions, and FMT methodologies across the included trials. Patient cohorts ranged from IBS, UC, fibromyalgia, PD, severe obesity, to MDD. These conditions differ markedly in their pathophysiology and in the mechanisms by which depressive symptoms arise, which limits the comparability and generalizability of the pooled results. Second, FMT interventions varied considerably in terms of administration route, dosing regimens, donor selection, and bowel preparation strategies. These methodological differences may influence microbiota engraftment and therapeutic outcomes. Third, depressive symptoms were assessed using heterogeneous outcome measures at follow-up time points ranging from 1 week to 12 months. This variability in measurement tools and assessment timing likely contributes to the statistical heterogeneity, and may affect the comparability of effect sizes. Fourth, most included RCTs were not primarily designed to evaluate depression as a main outcome. Only one study specifically enrolled patients with clinically diagnosed MDD, and it found no significant difference between FMT and placebo. In many other trials, depression was a secondary or exploratory endpoint, or participants exhibited only subclinical depressive symptoms. Consequently, the applicability of these findings to clinical depression populations is limited. Fifth, although subgroup analyses were comprehensive, confounding factors such as concomitant medications, psychological interventions, dietary influences, and genetic predispositions were not uniformly controlled across studies, potentially influencing treatment outcomes and introducing bias. Sixth, baseline recipient microbiota composition and antibiotic pretreatment were inconsistently reported and rarely analyzed in relation to FMT outcomes, which may substantially influence engraftment success and therapeutic efficacy. Finally, the observed clinical and methodological heterogeneity, combined with the relatively small number of trials in some subgroups, means that the synthesized results should be interpreted with caution.

## 5 Conclusion

This meta-analysis suggests that FMT may offer short- to mid-term improvements in depressive symptoms across various clinical populations, with a potentially greater benefit observed in patients with disorders of gut-brain interaction such as irritable bowel syndrome. However, the certainty of this evidence is constrained by substantial heterogeneity in study populations, intervention protocols, and outcome assessments, as well as by the fact that most included trials were not primarily designed to evaluate depression. The long-term efficacy of FMT remains uncertain, with some evidence indicating a decline in therapeutic effect over time. These findings highlight the need for high-quality, adequately powered randomized controlled trials employing standardized methodologies, consistent depression outcome measures, comprehensive microbiota analyses, and systematic reporting of confounding factors.

Future research should prioritize well-designed, adequately powered RCTs that specifically target populations with clinically diagnosed depression, rather than heterogeneous cohorts with mixed conditions. To improve comparability across studies, standardized FMT protocols are needed with consistent administration routes, dosing schedules, donor selection criteria, and bowel preparation strategies. Uniform depression outcome measures and longer follow-up periods should be employed to evaluate both short- and long-term effects. In addition, concomitant factors such as medications, psychological interventions, dietary influences, and genetic predispositions should be systematically assessed and controlled to reduce confounding. Integrate systematic baseline microbiota profiling and consider antibiotic pretreatment strategies, in order to optimize patient selection and maximize the therapeutic potential of FMT in alleviating depressive symptoms.

## Data Availability

The original contributions presented in the study are included in the article/**Supplementary Material**. Further inquiries can be directed to the corresponding author/s.
