# Supplementary material for: Clinical efficacy of fecal microbiota transplantation in alleviating depressive symptoms: a meta-analysis of randomized trials
Source: Front Psychiatry. 2025 Oct 6;16:1656969. doi: 10.3389/fpsyt.2025.1656969 (PMC12536323; doi:10.3389/fpsyt.2025.1656969)
Supplement: Supplementary file 1 [file DataSheet1.docx]

**Appendix 1:**

**Search strategies**

**PubMed:**

(depressive OR depression OR MDD) AND ("Fecal Microbiota Transplantation"[Mesh] OR "fecal microbiota"[tiab] OR "faecal microbiota"[tiab] OR "fecal transplant*"[tiab] OR "faecal transplant*"[tiab] OR "feces infusion*"[tiab] OR "faeces infusion*"[tiab] OR "donor feces"[tiab] OR "donor faeces"[tiab] OR "microbiome transplant*"[tiab] OR "microbiome transfer*"[tiab] OR "microbiota transplant*"[tiab] OR "microbiota transfer*"[tiab]) AND 2000/01/01:2024/12/31[dp] NOT (animals [mh] NOT humans [mh])

**Altered gut bacterial-fungal interkingdom networks in children and adolescents with depression**

**CINAHL (EBSCOhost):**

(depressive OR depression OR MDD) AND (MH "Fecal Microbiota Transplantation" OR "f#ecal microbio*" OR "f#ecal trans*" OR "f#eces infusion*" OR "donor f#eces" OR "microbiome trans*" OR "microbiota trans*") NOT (MH "animals" NOT "humans")

Limiters applied:

1. Published Date: 20000101-20241231

**Cochrane Library:**

("depressive" OR " depression" OR "MDD") AND ("Fecal Microbiota Transplant" OR "fecal microbiota" OR "faecal microbiota" OR "fecal transplant*" OR "faecal transplant*" OR "feces infusion*" OR "faeces infusion*" OR "donor feces" OR "donor faeces" OR "microbiome transplant*" OR "microbiome transfer*" OR "microbiota transplant*" OR "microbiota transfer*")

Limiters applied:

1. Title Abstract Keyword
2. Year first published: Jan 2000 – Dec 2024

**Web of Science:**

(TS=("depressive" OR " depression" OR "MDD")) AND (TS=("Fecal Microbiota Transplant" OR "fecal microbiota" OR "faecal microbiota" OR "fecal transplant*" OR "faecal transplant*" OR "feces infusion*" OR "faeces infusion*" OR "donor feces" OR "donor faeces" OR "microbiome transplant*" OR "microbiome transfer*" OR "microbiota transplant*" OR "microbiota transfer*")) NOT (TS=(animals NOT humans))

Limiters applied:

1. Publication Date: 2000-01-01 to 2024-12-31

**EMBASE：**

('depressive' OR 'depression'/exp OR 'depression' OR 'mdd') AND ('fecal microbiota transplant'/exp OR 'fecal microbiota transplant':ti,ab,kw OR 'fecal microbiota':ti,ab,kw OR 'fecal transplant*':ti,ab,kw OR 'feces infusion*':ti,ab,kw OR 'donor feces':ti,ab,kw OR 'donor faeces':ti,ab,kw OR 'microbiome transplant*':ti,ab,kw OR 'microbiome transfer*':ti,ab,kw OR 'microbiota transplant*':ti,ab,kw OR 'microbiota transfer*':ti,ab,kw)

Limiters applied:

1. Quick limits:[humans]/lim
2. Date limits: [2000-2024]/py

**Appendix 2:**

**Sensitivity analysis**


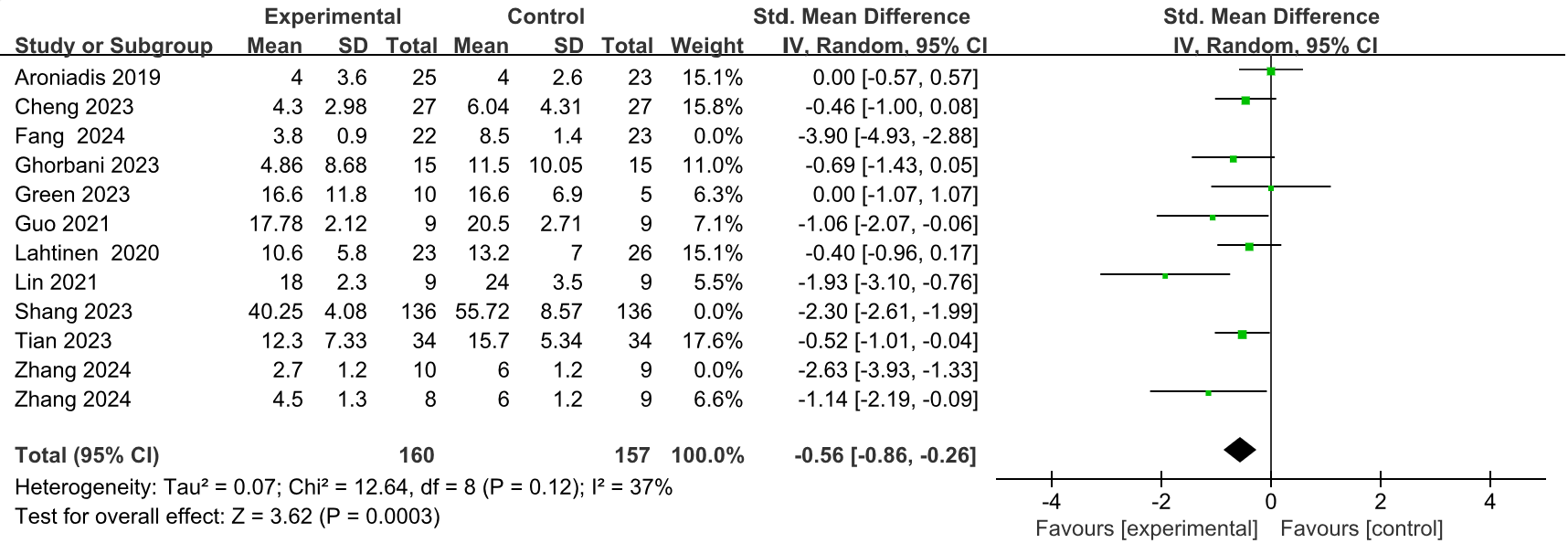


**Figure S1.** Sensitivity analysis of the effect of FMT on depressive symptoms


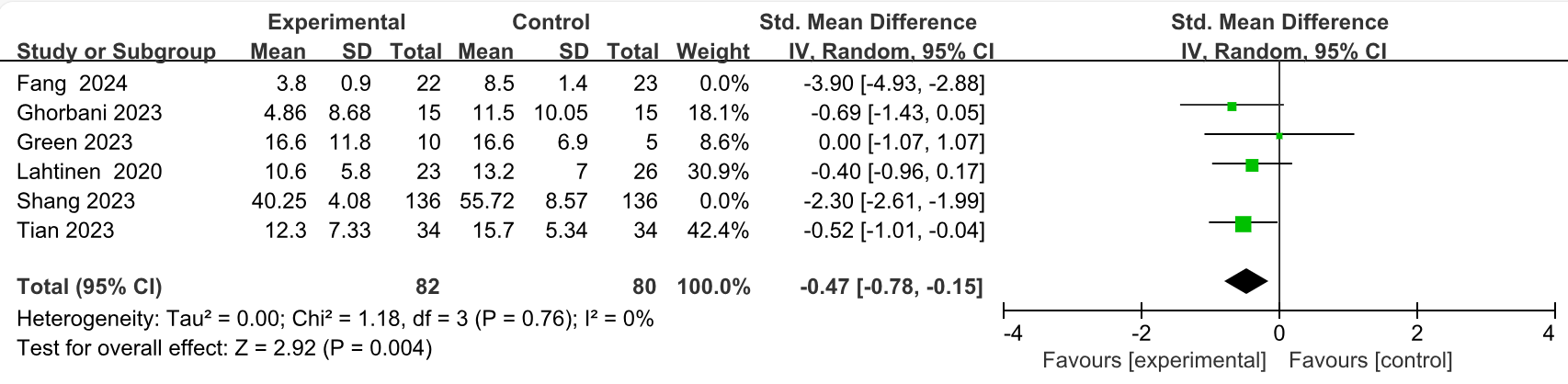


**Figure S2.** Sensitivity analysis of the effect of direct gastrointestinal FMT delivery routes on depressive symptoms
